# “From everything to nothing in a split second”: Elite youth players' experiences of release from professional football academies

**DOI:** 10.3389/fspor.2022.941482

**Published:** 2022-07-22

**Authors:** Thomas Ryan McGlinchey, Chris Saward, Laura Catherine Healy, Mustafa Sarkar

**Affiliations:** Sport, Health and Performance Enhancement (SHAPE) Research Group, School of Science and Technology, Nottingham Trent University, Nottingham, United Kingdom

**Keywords:** athletic identity, deselection, elite sport, football, interpretative phenomenological analysis, non-normative transition, soccer

## Abstract

Previous research has assessed the affects release from football academies has on psychological distress and athletic identity of players. However, there has been no qualitative research exploring players' experiences of the release process. This study retrospectively explored players' lived experiences of being released from a professional football academy, having completed a scholarship (from ages 16–18). Four male football players (age 21.6 ± 1.5 years) who had experienced release from professional academies participated in in-depth semi-structured interviews. Data were analyzed using Interpretative Phenomenological Analysis. Four super-ordinate themes were interpreted from the data: Foreshadowing release—“left out in the cold”, The process of release, Support during the process of release and New beginnings—“there's a bigger world than just playing football every day”. Players reported that their contract meeting was a traumatic experience, and they experienced psychological difficulties in the longer-term following release. Factors that compounded the players' release were: a lack of aftercare being provided by the players' professional clubs for their wellbeing, and a disuse of social support, which hindered their transition out of full-time football. Context relevant recommendations are made to help improve the release process for elite youth football players.

## Introduction

Of the 1.5 million boys who play organized youth football (soccer) in England, only ~180 will be signed professionally by a Premier League club, a success rate of 0.012% (Calvin, 2017). In 2011, 13,612 boys made up the professional football academy system in England. Despite this, 50% of all academy players leave the system before they are 16 (Premier League, 2012). Furthermore, ~98% of players awarded with an academy scholarship by English clubs at 16 are no longer playing in the top five tiers of English football at 18 (Calvin, 2017). These statistics indicate that a large number of boys do not realize their dreams of becoming professional senior footballers and are released from academies each year. Yet, with the focus of talent development football research on those who “make it”, this issue has received limited attention (Gledhill et al., 2017). Career transitions are normative or non-normative turning phases during an athlete's career (Stambulova et al., 2009). A normative transition is one an athlete typically expects to make, usually when exiting one life stage and entering another, such as the transition from youth to senior level sport (Wylleman and Lavallee, 2004). Release from a professional football academy is the termination of a player's contract and playing status with that club, and is an example of a non-normative career transition, whereby the athlete does not expect or anticipate the transition (Webb et al., 1998). Non-normative athletic career termination, such as release, can result in problematic transitions (Brownrigg et al., 2012). Factors contributing to problematic transitions include: when termination is involuntary, the development of a high athletic identity, when the athlete has limited control regarding the continuation of their athletic career and when the athlete perceives that they have failed to achieve their sporting goals and unfulfilled their sporting potential (Warriner and Lavallee, 2008; Park et al., 2013). When athletes' careers are terminated involuntarily, they are more likely to experience emotional disturbance and psychological distress, such as depression, anxiety, identity crisis or confusion, loss of self-worth or esteem, suicidal ideation, attempted suicide and trauma (Erpič et al., 2004; Warriner and Lavallee, 2008; Wippert and Wippert, 2008, 2010). Such negative psychosocial outcomes are dramatically reduced when athletes have autonomy over career termination (Park et al., 2013). Further, some research has highlighted that there may also be positive outcomes associated with transitioning out of elite sport (Knights et al., 2016). For example, Williams and MacNamara (2020) conducted retrospective interviews with ten former professional academy cricket and rugby players concerning their release, with players revealing during their time in the academy, they felt they had developed a series of key psychological characteristics that had supported their future success in other domains. Positive growth following players being “cut” from a provincial soccer team has also been reported previously, despite participants describing it as a traumatic event (Neely et al., 2018). However, the professional football academy system in the UK represents unique and complex psychosocial challenges for young aspiring athletes (Champ et al., 2020; Mitchell et al., 2020) and such positive outcomes have not been shown.

In 2012, the English Premier League and Football Leagues were at the forefront of implementing a new long-term strategy known as the Elite Player Performance Plan (EPPP) (Premier League, 2012). The plan was designed to increase the number of home-grown players participating within these leagues (Roe and Parker, 2016). Academies were audited as part of the process and were classified into four categories; with category 1 being the highest level (Premier League, 2012). Category 1 academies receive the most funding, are able to provide a wider range of support and are licensed to recruit and develop players from 5 to 21 years of age. Further, they are obliged to provide up to 8,500 h of coaching for players who enter the system at 9 and leave at 21 years old. A typical category one academy in England has a reported annual spend between £2.3 and 4.9 million (Larkin and Reeves, 2018). In contrast, the lower category academies receive less funding and provide fewer coaching hours (Webb et al., 2020). Despite strong commitment, dedication and personal sacrifices (Holt and Mitchell, 2006), players enrolled on a youth scholarship at a professional football club have not yet secured a professional contract, and therefore have very little control over their career development both short-term and long-term (Roderick, 2006). Not gaining a professional contract has previously been reported as a perceived failure by academy players, leaving them uncertain and fearful of this possibility (Sagar et al., 2010). Professional football culture and the academy culture environment has been described as ruthless and cutthroat in nature, with a high player turnover (Nesti and Littlewood, 2011). This can culminate with a player's release from a professional football academy, which has been associated with high levels of psychological distress, identity crises, and ineffective coping (Brown and Potrac, 2009; Blakelock et al., 2016, 2019).

Blakelock et al. (2016) assessed the likelihood of 91 elite youth footballers, aged 15–18 years, experiencing clinical levels of psychological distress following release. Psychological distress was assessed using the General Health Questionnaire-12 (Goldberg and Williams, 1988) which was completed at three time points: 7–14 days before, 7 days after, and 21 days after retain/release procedures. Released players experienced significantly higher levels of psychological distress than retained players, both 7 and 21 days after retain/release procedures. Furthermore, the levels of psychological distress reported by released players were reflective of severe psychological distress (Rai et al., 2012), at levels generally requiring service input from mental health professionals (NICE, 2011). In a follow-up study, Blakelock et al. (2019) also showed that the use of avoidance coping strategies following release was positively correlated with higher levels of psychological distress in elite youth footballers. These studies suggest that following release, players may be more likely to engage in maladaptive strategies to cope with their release and experience high levels of psychological distress. However, these studies only considered effects across a 6-week period at the time of release, and not the longer-term effects release could have on players. There is a need to explore how each individual actually makes sense of this experience, and how they cope with any longer-term psychosocial effects (Brown et al., 2018). Thus, the large number of boys being released from academy football (Premier League, 2012), coupled with clinical levels of psychological distress associated with release (Blakelock et al., 2016, 2019), indicates a necessity for a more detailed exploration of players' experiences and individual stories of the release process (Brown et al., 2018).

A major factor associated with problematic non-normative transitions, such as release, is the loss of a high athletic identity, resulting in an identity crisis (Lally, 2007; Warriner and Lavallee, 2008). Elite youth footballers are usually recruited into professional academies between the ages of 5 and 10 years (Green, 2009; Mitchell et al., 2020). This has raised concerns over the increased professionalization of football at a younger age and the potential detrimental impact this may have on players (Relvas et al., 2010). Fundamental to this increased professionalization has been early entry into academy environments, which places significant demands on young players and is viewed as their first entry into the football “profession”. Players can be formally associated with a club's academy from 9 years old and train between 2 and 4 times per week, as well as undertake competition on a weekend (Richardson et al., 2004). The prioritization of football, and the demands placed on players (i.e., attending training and fixtures regularly, and making sacrifices to the social and educational aspects of their lives, such as the day release program) can result in the creation of a strong athletic identity (Brown and Potrac, 2009; Mitchell et al., 2014). Research has suggested that the formation of a strong athletic identity in elite youth footballers can cause emotional disturbance when this identity is disrupted by release and hinders players adjusting to life away from full time football (Brown and Potrac, 2009). As so few boys sign professional contracts at 18 years (Green, 2009; Calvin, 2017), many may encounter identity crises and psychosocial problems when transitioning away from being a full-time footballer. The onset of such distress can be related to the concept of symbolic loss; through involuntary career termination, players lose the primary source of their identities and what has been the focus for most of their lives, causing an identity crisis (Brown and Potrac, 2009). However, identity disruption may form just one aspect of a player's experience during and after release. A more comprehensive exploration of individual's stories is necessary to discover personal experiences of release (Brown et al., 2018).

As far as the authors are aware, there have only been three studies exploring youth player release from professional football academies: from a clinical psychological perspective (Blakelock et al., 2016, 2019), and an athletic identity perspective (Brown and Potrac, 2009). A recent review of talent development in football highlighted players who were unsuccessful in their attempts to become professional footballers are under-represented in the literature (Gledhill et al., 2017). Therefore, it is vital that the understanding of players' perspectives and experiences of release, which remain largely unexplored, are enhanced further (Wilkinson, 2021). Previous research suggests that released elite youth footballers may encounter psychosocial problems (Brown and Potrac, 2009; Blakelock et al., 2016, 2019), but it is unclear how they cope with the long-term effects of their release, or how they view their experiences of being released. Thus, there is a need to explore the release process in greater depth, and through an idiographic approach, the individual stories of elite youth players who have been released. Therefore, the aim of the current study was to retrospectively explore players' experiences of being released from a professional football academy.

## Method

### Philosophical underpinnings and qualitative design

In terms of a philosophical orientation to this study, our underpinning epistemological approach was constructivist, from a theoretical standpoint of interpretivism. Constructivism asserts that people construct their own understanding and knowledge of the world through experiences and reflecting on those experiences (Honebein, 1996). Thus, we adopted a qualitative, interpretative phenomenological analysis (IPA) approach (Smith and Osborn, 2004). IPA has origins in phenomenology, hermeneutics, and idiography, and aims to understand individuals from a particular population's “lived experiences” through participants sharing stories, thoughts and feelings about their experiences of specific phenomena (Smith and Osborn, 2004; Langdridge, 2007). IPA involves a two-part interpretation, termed a “double hermeneutic”, whereby the participant is trying to make sense of their world and the researcher is trying to make sense of the participants making sense of their world (Smith and Osborn, 2004). As a focus of the present study was to explore each participant's individual story, IPA was deemed the most suitable approach due to its idiographic nature, which focuses on the particular and unique details of each case (Smith and Osborn, 2004). An idiographic approach is more explicit in IPA than in other approaches to qualitative research (Brocki and Wearden, 2006). Given that each individual's experience of release was likely to be different, it was hoped that IPA's focus on idiography would allow us to highlight both the divergent and convergent aspects of the participants' experiences of release. The aim of the present research then was not to describe objective reality, but rather to explore and understand each participant's view of the world as related to the phenomenon of interest (Smith and Osborn, 2004).

### Participants

IPA studies use homogenous purposive samples, to ensure the experiences of the most appropriate persons for the research question being addressed are sought (Smith and Osborn, 2004). Thus, following institutional ethical approval, four male footballers (age 21.6 ± 1.5 years, *M* ± *SD*) that had experienced release from elite level youth football in England were invited to participate. To meet the sample criteria for the study, participants had to have: (a) completed a 2-year scholarship (from ages 16–18 years) at a professional football academy, within categories 1–3 of the Elite Player Performance Plan [EPPP (Premier League, 2012)], and (b) been released from a professional football academy within the last 5 years to reduce any significant memory decay or bias (Pillemer, 2001). The interviews were conducted within a maximum of 3 years after the players' release. All participants provided informed written consent prior to participation in the study.

### Player biographies

We have provided a brief contextual synopsis for each participant, in alignment with IPA, for the reader to feel as close as possible to each player's story. The descriptions reflect our interpretations of the players' stories and footballing journeys. To protect their anonymity, we gave each player a pseudonym and their respective clubs and national teams were not named. Keith played for a category 3 academy whilst the other three players played for the same category 1 academy.

**Keith** is 23. He signed for a professional academy at age 13 before completing his 2-year scholarship. He earned a 1-year professional contract during which the club won a lower division title. He was then released at age 19. Keith moved into semi-professional football and was placed on the national “C” team contingency list—international recognition for semi-professional players under 23. Keith continues to play semi-professional football whilst working as a bricklayer, having recently completed a night-college course. He has a child.

**John is 22**. He signed for a professional academy at age 8 before coming out of the academy a year later by choice. Having re-signed for the same club a year later, he completed a 2-year scholarship, gaining international recognition with his national team's under 19's in his second year. John was then offered an extended scholarship for a further year due to injury before being released at age 19. After unsuccessful trials at several professional clubs, John signed for a full-time semi-professional club. Following numerous knee injuries that required four operations John had to quit playing football. John is now an estate agency valuator actively pursuing a career in the fire service.

**Arthur is 22**. Arthur and John played for the same professional club in the same age group. Having signed for a professional academy at age 8, he completed a 2-year scholarship and a 2-year professional contract at the club. During his time at the club, he earnt international recognition with his nation at U16, U17 and U18 level. Following two hip operations Arthur was released by the club at age 20. Having had one unsuccessful trial at a professional club he signed for a part-time semi-professional team. However, due to a hip injury, he quit playing football. He is now an ambulance technician.

**Ben is 21**. He signed for a professional academy at age 9 before completing a 2-year scholarship and a 1-year professional contract. Ben was then released at age 19, before unsuccessfully trialing at numerous clubs, both in the UK and the USA. He then signed for a full-time semi-professional side before moving into part-time football when his previous club had financial issues. During his semi-professional career he has gained senior international recognition for his national team. He currently works in a warehouse but maintains a strong desire to re-enter full-time football.

### Procedure

Single retrospective, in-depth semi-structured interviews were conducted individually with each participant. Interviews lasted between 52–81 min (*M* ± *SD* = 71 ± 13 min). Interviews took place in locations mutually agreed with the participants, where they felt relaxed and comfortable to share their experiences of release with the interviewer. As recommended by Smith and Osborn (2004), a semi-structured interview guide was designed, to facilitate a flexible interview format. Adopting a semi-structured interview format created a dialogue between interviewer and interviewee, allowing both the participant and interviewer to discuss topics that arose (Smith and Osborn, 2004). This aligned with IPA as it allowed the interviewee to speak about their feelings and experiences in great depth, resulting in richer data being generated. The participants were initially asked general questions about themselves to build rapport and ease them into the interview process. The main topics of the interview involved asking participants to describe their experiences of the period leading up to, the period around the point of and the period following release, to get to the heart of the players' lived experiences of the release process; for example, “Can you tell me about the period leading up to your release?” and “Can you tell me about the day you were released?”. Each interview was audio-taped *via* a Dictaphone before being transcribed verbatim, yielding 107 pages of single-spaced text.

### Data analysis and interpretation

Guided by the principles of IPA (Smith and Osborn, 2004), data analysis began with re-reading the interviews several times, to become as familiar as possible with the data. Then, initial annotations were made, identifying anything of interest or significance, before being transformed into concise phrases to capture the essence of themes. Here, attention was focused on the language used by the participant, including identifying repetition of particular words and the way that the account was expressed (Nizza et al., 2021). Also, we tried to move beyond what was explicitly said to gain a deeper understanding of the meaning that was attached to what was being discussed. Following this, connections between themes were searched for and identified, so they could be grouped together into superordinate themes. Lastly, the superordinate themes were checked with the transcripts to ensure they reflected the actual words of the participants. The whole process, from initial notes to developing superordinate themes, was conducted for each participant separately. Finally, a cross-case analysis was conducted, in which themes and superordinate themes for each participant were assessed for similarities and differences. Identifying higher order concepts made it possible to link the participants' experiences, yet still reflect divergence and maintain the idiographic focus that is central to IPA (Nizza et al., 2021). As IPA arose from a phenomenological tradition that seeks well-grounded descriptions in the analyzed text, we strived to achieve this by showing rich descriptions from the interviews through quotes (Smith and Osborn, 2004).

### Methodological rigor

We aimed to follow the four quality indicators of a good IPA paper (Nizza et al., 2021). This included constructing a compelling, unfolding narrative, highlighting the existential accounts provided by the players, a close analytic reading of the players' words and attending to convergence and divergence in the players' stories (Nizza et al., 2021). We endeavored to ensure that we considered both the phenomenological descriptive part and the interpretative freedom of IPA (Smith and Osborn, 2004). To achieve these aims, the first author analyzed all material independently; with the second and third authors having the role of “critical friends” (Marshall and Rossman, 2015). The role of the “critical friend” is not to try and achieve consensus, or agreement, as this does not guarantee rigor or mean the “truth” has been found, but rather to encourage reflexivity by challenging one's construction of knowledge (Patton, 2015). Thus, the different perspectives offered by critical friends serve to challenge and develop the interpretations made by the researcher as they construct a coherent and theoretically sound argument to support the case they are making in relation to the generated study data (Smith and McGannon, 2018). Furthermore, the first author kept a record of interpretations of the data throughout the interviewing and analysis process, to allow reflections over time and reflexivity on our own interpretations (Tracy, 2010). Throughout the research process we tried to remain aware of our biases. The first author was a youth player in a professional football club academy from age 5–14, before being released. Therefore, it is unrealistic to think there may not be some biases toward the academy experience and release. However, the constructivist paradigm emphasizes that research is a product of the values of researchers and cannot be independent of them (Honebein, 1996). Further, IPA draws heavily on the philosophical work of Heidegger's (1962) hermeneutic phenomenology, which suggests we cannot ignore our biases, but rather interpret experiences based on our cultural and historical background - our prior understandings. These considerations allowed us to be reflexive throughout the research process (Tracy, 2010).

## Results

Themes have been organized into a coherent, unfolding narrative that is representative of the participants' lived experiences of their release. The results detail the players' release as a process; beginning with the lead up to their contract meeting, the point of their release and the period of time after their release. Four superordinate themes were identified and developed to reflect the process of release: foreshadowing release—“left out in the cold,” “the process of release,” “support during the process of release,” and new beginnings—“there's a bigger world than just playing football every day” The resulting list of superordinate and subordinate themes is presented in Table 1. We have strived to highlight convergence and divergence between players' stories, with quotations—selected for their richness—used to illustrate these themes (Nizza et al., 2021).

**Table 1:** Table of themes identified from the interviews.

**Superordinate themes**	**Subordinate themes**
Foreshadowing Release—“Left Out in the Cold”	• The nature of professional academy football • Marginalized by the club
The Process of Release	• The contract meeting—“a traumatic experience” • Psychological difficulties in the aftermath • Accepting release
Support During the Process of Release	• Club aftercare • Support from significant others • Seeking professional support
New Beginnings—“There's a Bigger World than Just Playing Football Every Day”	• Moving on • Growth after release

## Foreshadowing release—“left out in the cold”

Through our interpretations, we felt the unique environment of professional academy football influenced the players' experiences of the release process. Contributing factors that underpin this unique environment were the uncertainty players felt in relation to their place within the academy and being marginalized by their clubs in the build up to their release.

### The nature of professional academy football

All the players expressed how unpredictable their footballing careers were, and the ups and downs they experienced. Keith summed up the unpredictable nature of football, and the “emotional rollercoaster” players experience day-to-day:

“… with full time football you don't know.. every day is different, your emotions every day are different, you're physically different, you can have a great training session, you can have a shit one.. you don't know what tomorrow is gonna bring. Whereas at work you know you're gonna go work and you know what you're gonna do.”

Players talked repeatedly about the element of luck involved, both in terms of injury, and getting the break they needed, to go onto a career in professional football. This unpredictability contributed to the players' experiences of release, as they were uncertain of their futures within football. Adding to this, all the players highlighted a lack of control and feelings of helplessness whilst pursuing their dreams of becoming professional footballers in their respective academies. This included the players' perceived inability to change the opinions of coaches and directors of football. Keith spoke candidly about his pursuit of becoming an established professional footballer:

“I regret like not working harder but the carrot didn't dangle, if that makes sense? Like, they didn't give me that incentive. As if like.. ‘cos I worked hard, but there wasn't that incentive, do you know what I mean? Whereas if they'd give me an incentive like, ‘oh if you carry on the way you're going you'll be playing first team or you'll get your chance'. It was just kind of like, just not bothered, like if I go home or stay, no matter what I was doing, like in my head, no matter what I was doing I weren't getting noticed so.. you end up kind of sacking it off in the end.”

Keith spoke about how that subsequently made him feel:

“Shit. Like that's what I mean, you end up hating it don't you, there's no drive, there's no like, love for it, you just turn up, you do it, get through it and then just go home.. because you know even if you have a worldie training session, nothing's gonna matter.”

Throughout his interview, Keith openly reflected on not feeling good enough to establish himself at senior first team level, but also discussed his interpretation of his situation—whatever he did was not going to get him noticed—a point echoed by Arthur. Keith and Arthur's insights reveal why players may not act in a volitional manner or positively self-regulate if the perceived reward for these behaviors is not there.

### Marginalized by the club

All the players talked of being marginalized by their club and coaches before the point of release. Arthur recalled one such instance at training in the lead up to his contract meeting:

“I don't think personally they treat you as one of their players anymore, they treat you as just.. ‘oh he's turned up to training again'.. so.. there was a few sessions where they'd do a small sided tournament.. and I'd just be a floater.. or I remember once, I turned up to training and they didn't even know that I was coming, and they didn't even put me in any of the teams.. erm.. and that was training with the first team as well, so I was looking forward to it, and then I walked up to the pitch and they said, ‘oh Arthur, you're not in any of the teams'.”

This public embarrassment and humiliation Arthur recalled in front of his teammates and first team players left him feeling angry, hurt, and ashamed. The marginalization by their clubs reported by the players is one way in which their release was compounded, adding to the negative emotions the players experienced during their time at the club and following their release. It is interesting to note whether this marginalization was particularly evident to the players at the time, or in trying to make sense of their experiences of release during the interviews, they were looking back for clues of what was to come. However, all participants noted instances of either themselves or teammates being marginalized and “left out in the cold”, which suggests it is engrained within the game of football. This was typified by Arthur highlighting conversations the club had with his agent without his knowledge about the probability of his release, whilst Ben spoke of being left out of matchday squads in favor of trialists.

### The process of release

Through the players' stories and experiences of release, we interpreted their release as a process, rather than a singular event. While all players talked of an immediate, emotional response to being released after their contract meeting, for John, Arthur and Ben, their most severe psychological difficulties were experienced some time after the event of release.

#### The contract meeting—“a traumatic experience”

All the players revealed being released was a traumatic experience and highlighted feelings of sadness, pain, and anger. The players were all released in the same way: their team gathered in a changing room and one-by-one, they were called into their contract meeting—one of the common aspects of all the players' experiences of release. When the players discussed their release, the pain and emotion was evident as they recalled the events, which was typified through John and Keith's repeated use of the word “horrible” to describe their feelings around the time of their release. Players recalled feelings of anxiety, doubt, and uncertainty the wait for their contract meeting caused. John recalled the emotional trauma of his contract meeting and the subsequent aftermath:

“..they said that I'm getting released.. ‘but (professional club) really want you, they're gonna literally.. like they've got a two year contract, and you've just got to play well'.. then I'd hardly even spoke to my Mum and Dad, literally had to pack my bags and pretty much go the next day.. I thought I was fine, I didn't think I was bothered about it at all.. then I got to (professional club) and it literally all like, hit me, and I was thinking like.. I was literally gutted. I just remember like literally.. I think I just sat and cried in the room in (city of trial club). It was horrible.”

Keith recalled how he felt immediately after being released in his contract meeting:

“Yeah, it was.. emotional. Erm.. obviously you hold it all in but you're sitting there and it's like.. I've been there for five years, you know like the kit man, all the lads, the coaches and you literally get told no, and it's within ten minutes, you've got all your stuff and you're out and you never see them again.. so it's just kind of like from everything to nothing in a split second.”

Through interpreting the players' reflections, this appears to be a defining aspect of the release process, and why it may cause such distress to players. In one moment, they are full-time footballers, which their identity is inextricably linked to; the next, this identity has been suddenly taken away, causing emotional harm and leaving the players questioning their place in their lifeworld. This shattering of identity was poignantly captured by Keith—“from everything to nothing in a split second”—and highlights the existential accounts the players gave of their release experiences.

#### Psychological difficulties in the aftermath

John, Arthur, and Ben reported experiencing psychological difficulties in the aftermath following their release. John reflected on the psychological effects of his release:

“To be fair, I've let it go now, and I think.. I think at the time I weren't that bothered. Obviously, I broke down at (professional club he trialed at the day after release) once, but that was like literally not even touching it.. my knee just kept swelling up, kept swelling, kept swelling up, I knew.. I knew I needed an operation like straight away, but I knew I had to find a club first. Then I was just playing on the swelling for about six months.. then it broke down and.. I had a massive operation, I knew about my knee, I knew I couldn't really.. I could have gone back to football but I knew mentally I couldn't.. then after that, I literally just remember.. I was like, so down. I had counseling and I just remember I had like, four.. four-hour counseling sessions and I must have just cried for about 45 minutes of each. Yeah, I felt well better after… I think it makes you a better person now ‘cos you're so like resilient to most things.”

John's words encapsulate his experience of release as a process, rather than just a one-off event. The quote begins by him playing down his psychological difficulties before he completely opens up by initially talking about breaking down at his trial club the day after he was released in his contract meeting. However, this emotional trauma “was like literally not even touching it,” in terms of how bad it would get. Over 6 months after his release, and in combination with a career threatening injury, John spoke candidly of his severe psychological difficulties and seeking professional support. Finally, he discusses the increased resilience he now possesses following his experience of release. Evidenced by the pain and emotion they displayed during their interviews, both John and Arthur expressed the most emotional and psychological turmoil of all the participants following their release. This may be due to their release being coupled with career-threatening injuries. This finding is salient, as players who are released either due to, or with an ongoing injury, may be more susceptible to experiencing higher levels of psychological distress than players without ongoing or career-threatening injuries. Furthermore, Arthur and John's injuries were not recognized immediately as career ending, and they both went through arduous periods of pain and rehabilitation believing they would be able to continue playing.

Ben recalled the effects release had on him:

“I won't lie to you, yeah I did get down after I left. Like, properly.. like I'm not gonna sit here and try and tell anyone it doesn't affect you, it affected me more, like.. a year after.. or like, 6/7 months after. When I hadn't been playing, the frustration set in and I was telling my parents, ‘I don't care about football anymore'. I was literally.. I was so done with football.”

Also, Ben expressed the psychological difficulty he experienced was partly due to financial worry:

“Depression.. this is like purely financially as well, yeah. So.. basically, I'd been released for a little while.. I earnt a decent amount of money for my age.. when that stops happening, yeah, that's when you feel it. I promise you now. Honestly, not having the freedom that.. of having money.. like, literally man, that ruined me.. you're literally just watching your pocket.. plus my car, this that, like, you start adding everything up and it just.. like, it grows.. the burden of it all grows and.. that's when I started to feel down.”

John, Arthur, and Ben appeared to experience severe levels of psychological distress following their release, encompassing feelings of depression, identity crisis and confusion, loss of self-worth and esteem, sadness, pain, and anguish. Furthermore, these three players endured delayed psychological difficulties between 6 months and 1 year post their release for a prolonged period of time. After the point of release, these difficulties seemingly worsened with time as the realization of their release sank in. In contrast, Keith did not report experiencing distress to the severe levels of the others in the longer term, instead highlighting the positive aspects of being released; “I think it probably had more of a positive effect in the end because I ended up enjoying it (football) again.” Keith's quote highlighting his positive response and lack of psychological difficulties in his longer-term response to his release demonstrates the divergent experiences and responses to release between players. From the players' retrospective interviews, it was clear to see that each individual's experience of the release process was bespoke and had nuances, and each player responded differently.

#### Accepting release

One such individualized aspect the players talked of was varying levels of acceptance over their release, and how this changed over time. Arthur and John took the longest to accept their release, most likely due to long-term injuries they were suffering with, whereas Ben accepted his release sooner. All participants accepted their release in the longer term. Keith took ownership over his release, which resulted in him accepting the club's decision almost immediately following his release:

“I think ‘cos in the back of your mind, you know you're not good enough to be there, especially going into League 1 as well, at the time, so I think I kind of accepted it, like, in my head without knowing I'm accepting it, if that makes sense.”

The ownership and acceptance of his release allayed anxiety Keith had when informing others of his “perceived failure” to earn another contract: “Not that bad to be honest. Just it is what it is.. you've got to be honest with them. There's no point lying or.. saying.. or blaming other people. You just say it how it is…”. In contrast, Arthur struggled to accept his release initially and discussed whether his psychological difficulty was related to his release or his injury...

“Erm.. yeah just ‘cos of my injury really, ‘cos I knew if I hadn't been injured, I would have been made it as a professional footballer or a first team player.. erm.. so it was always in my mind like ‘oh what if I didn't get injured?' Or, ‘what if I.. what if I.. just didn't train some days?' Or, ‘what if I just rested it some days?' Or, ‘what if I went.. seen the physio earlier, before my injury?' or something like that.”

Arthur subsequently recalled how that made him feel: “Err.. upset… I felt just like… alone all the time, especially with being away from the club.. any club really, and just being around the banter and stuff.”

As previously highlighted, Keith expressed far fewer psychosocial difficulties in comparison to the other participants following release. It appeared that Keith experienced less difficulty transitioning away from full-time football due to his ownership over release and acceptance of not being good enough for first-team football. From our interpretation, Keith demonstrated a greater emotional maturity than the other participants when talking about and reflecting on his release. This is possibly due to being older than the other players, but also owing to the fact he had a young family when he was released, thus ‘grew up' earlier. In contrast, Arthur lacked acceptance and ownership over his release, and subsequently experienced severe psychological difficulties. His lack of ownership appeared to come from a belief his release was out of his control, due to a major injury, and a belief he would have become an established professional player without this. Keith and Arthur's contrasting levels of acceptance of their release again highlight the divergent experiences players had with their release.

## Support during the process of release

Players discussed aspects of support throughout the release process and how they perceived this to affect their psychological and emotional response to release.

### Club aftercare

All the players reported receiving no support for their wellbeing from their club following their release. This left them feeling sad, angry, and isolated. Arthur recalled the lack of support provided by the club:

“The club kind of just leave you to it.. which is.. hard because you'd been there for so long. Then they just brush you aside like you're nothing.. which is why.. that's how you know it's more of a business more than.. more than anything.. erm.. so I had to just try and find it myself.. and just work off my own back really.”

Keith echoed a perceived lack of support provided by the club:

“..they give it the old token ‘oh if you ever need anything, give me a call', but you never would. It was just kind of off you go and that was it. Just sort yourself out.”

All the participants expressed feelings of anger and betrayal, that clubs they had dedicated their lives to for so many years provided them with such little support, adding to the difficulties they experienced during the transition out of professional football. During all the interviews, the lack of perceived aftercare provided by clubs was noticeably a bitter pill for players to swallow and anger was evident in their voices. Despite all the players highlighting the need for support in dealing with the emotional and psychological problems that occurred following their release, Keith's quote revealed a reluctance to reach out to his former club for support, which was echoed by the other players. It was interpreted that former clubs who the players perceived had caused their suffering would be the last place they would seek support from.

### Support from significant others

All the players reported that emotional support was available from their parents but highlighted not wanting to burden their parents with their emotional suffering and difficulties. John spoke of the guilt he felt when the realization of not being able to play again because of injury sank in, due to the sacrifices and commitment his parents had made throughout his football career:

“The amount they sacrificed, I was more gutted for my Dad that I weren't gonna be able to play again. Then.. it all just

got way too much and I just broke down ‘cos of that. Then.. then I realized I weren't gonna be able to play football.”

It was evident during the interview that John was very emotional when talking about the role his parents played in supporting his football career, and one of the factors that distressed him the most following his release was the sadness and guilt he felt at not being able to “repay” his parents' sacrifices by carrying on playing. The lack of social support players sought from family and friends possibly added to the sense of isolation the players felt after being released, compounding the distress they experienced. For John, Arthur, and Ben, by bottling up their emotions and not sharing their struggles with significant others, the emotional turmoil they suffered increased.

All participants highlighted the positive team spirit of their respective youth teams and the strong bonds they developed with other players, describing the team as “like a family.” When players and their teammates were released, the participants highlighted the “shared experience” they all went through. Arthur commented:

“My age group.. I think most of them got released the year before.. which was hard for me ‘cos it kind of left us.. like me on my own.. with the younger players.. erm.. but yeah there was a few that got released at the same time as me. We was all going through like a difficult time, which was.. well it was good that we could speak to each other about it and stuff.. erm.. and they.. yeah they were just going through the same thing really.”

Throughout the interviews with the players, it became apparent that being released was a “shared experience” with teammates. The players within the present study viewed teammates who had also been released as a valuable support network, especially when compared to their friends outside of football, who they felt found it hard to relate to what they were going through.

### Seeking professional support

Both Arthur and John actively sought help themselves to deal with the psychological and emotional difficulties they were experiencing after being released. Arthur talked about seeking support from the Professional Footballer's Association (PFA):

“Five months after.. maybe six months after.. I'd left. Erm.. I just remember one day I was just too down and I wasn't doing anything, I wasn't moving out my bed, I wasn't talking to anyone, I wasn't.. I didn't want to be like.. productive or anything. So.. I just.. they'd always told you at football, if

there's anyone you want to speak to then you can ring the PFA, and I thought I'll just try it.. and it was.. erm.. they sorted me out with someone.. ASAP, like they gave me someone.. I think the next day to speak to, which was good. And I just seen him weekly.. and then as it got better.. you know, you just see him monthly and then you're just seeing him like every other month and then.. so I did that for about.. a year and a half or so.. erm.. I think if I hadn't spoke to anyone, I would have just.. I woulda been just too sad all the time… I spoke to him regular.. so.. and he was just helping me get through the process and stuff.. erm.. so through that time, it was hard, it was difficult, but when you're speaking to someone and letting them know how you feel, and letting your feelings out.. because when you're in a.. like a man environment, in a football environment, nobody really speaks to each other about your feelings like that.. so over time it was difficult but it was getting better.”

Although Arthur accessed the counseling services available through the PFA for its members, John accessed an independent counseling service over a year after he was released whilst undertaking physiotherapy for his long-term knee injury. Arthur felt the PFA provided him excellent support in helping him cope with his psychological difficulties. Similarly, John found the experience of talking to an independent counselor very beneficial in coping with his distress. The benefit players experienced from seeking professional support is salient, and highlights the positive impact aftercare can have on released players if provided by clubs.

## New beginnings—“there's a bigger world than just playing football every day”

Players discussed how they moved on following their release and some of the positive psychosocial outcomes that resulted from the healing process.

### Moving on

All the players felt they could have been better prepared by their clubs for release, but there were contrasting views on how this could be improved. Ben discussed the difficulty of preparing players for being released:

“No one really.. is ever gonna sit down and like.. have a meeting with you and be like ‘ah okay, this is what it's gonna be like when you're released', ‘cos no one wants you to be released, so they kind of avoid the subject in all cases, and if it happens.. ah, you know what, it's unfortunate. It's not like,

they have like meetings where.. ‘okay, what do you want to do outside of football?'.. It's not like school, ‘oh what do you want to do when you leave school?' Like, it's not like that at all. Like, school.. everyone knows they're leaving school, but football, they kind of avoid the fact that people do get released and the shit side of it.”

Ben's quote highlights the difficulty for clubs in trying to prepare players for being released. Possibly providing a solution to this problem, Arthur expressed a desire to try and help current youth players prepare for the possibility of being released:

“Yeah I said that to the person from the PFA. Obviously he.. I spoke to him about my like stuff, and my feelings and stuff, and he said to me ‘oh why don't you go and.. go into clubs and talk about that?' And I said, ‘yeah I would like to do that, I just need to look into it a bit more'. But if that opportunity came up, I'd definitely do that.. ‘cos the word needs to get out more.. and more people need to know and youngsters.. like now I know a few players that have just been released this season and I can see it in their face, like.. how down they were but they don't say anything about it. But yeah, I'd definitely do that if I could.”

This quote highlights the cathartic nature of both Arthur's counseling experience and the research interview. Initially, Arthur would not have been able to talk about his experiences because of the initial emotional trauma, but over time he has demonstrated his openness to new possibilities.

### Growth after release

A positive that all participants talked about during their interviews was an increased psychological resilience following their release, stemming from an enhanced sense of personal strength. John stated: “You literally work so hard for everything that literally gets ripped away from you, I think to go through all that.. I don't think there's anything I'll struggle with mentally after that.” Both John and Arthur were released due to injury, which ultimately led to their retirement from football. Upon coming to terms with their release, the players talked of looking forward, and their plans for the future. Arthur stated:

“..but now I look at it and think.. like I do miss football but.. it's only a short term thing.. erm.. and like the world's massive, like.. there's a bigger world than just playing football every day and.. there's so much more to do, and so much more to see, and.. like I said football's restricted, you can't do certain things, like with your friends and stuff. You miss

quite a few.. like just social time.. which I can do now and I think, you feel more like, free.”

Arthur's quote, “there's a bigger world than just playing football every day,” highlights the existential accounts given by the players regarding their experiences of the release process. For Arthur, having a newfound freedom, from both a high, foreclosed athletic identity and the “all-consuming football environment”, has allowed him to develop a new outlook on life and view of his personal world. Following the healing process of coming to terms with being released, and in Arthur and John's case, not being able to play football again, the participants have begun to construct new identities for themselves. Whereas the other participants have, at the least, constructed partial new identities, Ben still clings to his identity as a footballer, and is determined to re-enter full-time football. If he does not re-enter full-time football, this may be problematic to his long-term development, as holding on to his athletic identity may reduce his ability to build a new identity, potentially putting his life on hold.

## Discussion

This study was the first to qualitatively explore players' experiences of release from professional football academies. The four players, John, Arthur, Ben, and Keith all interpreted their release differently, but all viewed the release itself as a traumatic experience. For instance, all four players recalled almost identical contract meeting experiences: being gathered in the changing rooms and called one-by-one to their contract meetings, before returning to the changing room having learnt their fate, which caused them to feel shamed, embarrassed, and humiliated in front of their teammates.

A salient finding of the present study was the interpretation of release as a process, rather than a singular event, through the players' stories and experiences. This finding supports previous research that sport retirement is a process as opposed to an isolated event and extends knowledge in this area as release is a non-normative transition relative to athletic retirement at the end of an athlete's career (Taylor and Ogilvie, 1994). This process began with release being foreshadowed by the marginalization of players in the lead up to contract meetings. We interpreted the players immediate responses to release after their contract meeting as emotionally traumatic—characterized by the players' repeated use of the word “horrible”. Although some caution needs to be applied when associating players' release with trauma, in psychology, trauma is based on a person's subjective interpretation of an event rather than the objective characteristics or consequences of the event (Joseph, 2011). A defining aspect of trauma is the event causing physical or emotional harm, leading people to challenge assumptions about themselves and the world in which they live (Janoff-Bulman, 1992). The contract meeting causing emotional harm was clearly demonstrated by John, and Keith candidly captured how his release led him to challenge all his prior assumptions about himself and his world—“from everything to nothing in a split second”.

In the longer term, John, Arthur and Ben experienced psychological difficulties due to their release, including feelings of depression, anxiety, identity crisis and loss of self-worth, low esteem and confidence, which is supported by previous research (Blakelock et al., 2016). For these players, their most severe psychological and emotional distress was delayed, and experienced some time after the event of release. This supports the idea that release was not just a one-off moment, rather a process, for these players. It appears the effects being released has on players may not be experienced fully at the time of release, but after a delayed response—years in some cases, extending previous research (Brown and Potrac, 2009; Blakelock et al., 2016, 2019).

The individualized nature of the experience of the release process was also evident in the divergent psychological and emotional response to release between players. Specifically, it appeared as though levels of acceptance over their release affected the players' psychological and emotional response, as demonstrated by Keith and Arthur. As Keith took ownership over his release due to a perceived lack of ability, he may have begun preparing psychologically for release, thus appraising it as less harmful and threatening, reducing distress (Blakelock et al., 2019). This appraisal of release was also helped by the fact he transitioned into full-time non-league football, adding to his sense of perceived control of his situation. This is an example of problem-focused coping that has previously been shown to reduce psychological distress in released youth footballers (Blakelock et al., 2019). He also stated positive psychosocial outcomes as a result of his release such as re-finding his love for football. In contrast, Arthur perceived he had very limited control over his release, due to a long-term injury, and was thrust out of professional football unexpectedly and suddenly. This may have led him to appraise release as particularly harmful, increasing the psychological distress experienced (Blakelock et al., 2016, 2019). The wishful thinking and denial Arthur discussed in relation to accepting his release are indicative of avoidance coping strategies, which have been shown to increase psychological distress in released youth footballers (Blakelock et al., 2019).

A salient finding from the present study was the severe psychological difficulties experienced by those players who perceived that their release was injury related. Both Arthur and John suffered long-term injuries before they were released and reported experiencing severe levels of distress for a long time after their release, culminating in their forced retirements from football. This supports previous findings of the psychological difficulties athletes encounter through injury enforced retirement (Wippert and Wippert, 2010; Park et al., 2013; Arvinen-Barrow et al., 2017). The perceived lack of control both injured players had in relation to their release might be a factor in the magnitude of the psychological distress they experienced. Previous research suggests that athletes are more likely to experience emotional disturbance and psychological distress when their career is terminated involuntarily than athletes who have autonomy over career termination (Warriner and Lavallee, 2008; Wippert and Wippert, 2008; Park et al., 2013). This is consistent with findings from the present study and extends previous research into the area of elite youth football.

Despite being an obviously negative experience for players, the psychosocial problems related to release appeared to be compounded for players in this study by a series of factors. Firstly, the unpredictable nature of the professional football academy environment may not be conducive to a healthy transition out of academy football. Players reported feelings of helplessness, and a lack of control within the academy environment, which compliments previous research (Roderick, 2006; Sagar et al., 2010; Nesti and Sulley, 2013). This was typified by Keith's quote referring to his amotivation in the lead up to his release; a psychological state in which a person lacks either self-efficacy or a sense of control when striving to attain a desired outcome (Ryan and Deci, 2000). Conforming dedication to football has previously been stated as a psychosocial trait associated with progression to senior professional football (Holt and Dunn, 2004). However, as Keith outlined, if the reward for displaying these behaviors is not perceived to be there, players may not act in a volitional manner or display this conforming dedication. All the players also highlighted instances of being marginalized before being released, which foreshadowed the players' fates. After they were released, whilst they were still training with their club, they were “left out in the cold”. This public embarrassment and humiliation in front of teammates the players discussed led to feelings of hurt and shame. Shame is a painful emotion that is associated with feelings of inadequacy and the perception that one's entire self is a failure (Lewis, 1992). Previous research has highlighted that being released is viewed by players as failure and they are highly fearful of this outcome (Sagar et al., 2010). This demonstrates how the immediate emotional trauma of release was exacerbated for the players in the days following their contract meeting; being dehumanized and made to feel worthless by clubs they had dedicated large portions of their life to.

Another factor that likely compounded the psychological difficulties experienced by the players was reduced social support following release. Social support refers to “social interactions aimed at inducing positive outcomes,” (Bianco and Eklund, 2001, p. 85) with previous research showing its importance in positive sporting career transitions (Park et al., 2013). Players reported receiving limited aftercare from their professional clubs following their release, leaving them feeling hurt, angry and with a sense of betrayal. The lack of aftercare provided by clubs contributed to the problematic transitions experienced by the players, which is supported by previous research (Brown and Potrac, 2009; Wilkinson, 2021). It is important to note, despite the perceived lack of support the players were provided by their clubs, Keith commented on the reluctance players might feel in reaching out to a club they were released by for support. There may be a disparity in the perceived available and received support to players, which typically refers to the frequency with which an individual has received supportive resources during a specific time frame (Gottlieb and Bergen, 2010). This suggests further discussions around what support released players would benefit from is warranted and highlights the challenges clubs face in terms of release aftercare.

Players did not seek support from their parents due to feeling guilty about letting their parents down and not wanting to burden them with their problems, despite perceiving that their parents' support would be readily available to them (Freeman, 2020). This seemed to lead to John, Arthur, and Ben bottling up their emotions, which may have been a factor in the longer-term psychosocial problems they experienced. Previous research has highlighted the benefits of players using their parents as a source of support during the deselection process (Neely et al., 2017). However, the players in the current study were older than the athletes in the Neely et al. (2017) study - a key difference as to why the parents of the players in the present study were not as involved in their son's careers. This was evident through the players not needing tangible support such as transport to training and games, and in some cases living away from their home in “digs” (Freeman, 2020). Interestingly, the players reported sharing the experience of release with their teammates, who were viewed as a positive source of support both before and after the players were released (Neely et al., 2018).

A novel finding of the present study was the positive psychosocial outcomes in the players following their release, which have not been previously shown in the unique context of elite youth football. Although release was described as a negative experience, all the players discussed positive personal development following their release. The openness to new possibilities outlined by Arthur, and the perceived increase in personal strength John noted, are characteristics of posttraumatic growth; defined as experiencing positive change as a result of the struggle with major life crises (Tedeschi and Calhoun, 1995). All the players spoke about aspects associated with posttraumatic growth, including an appreciation of life, and relating to others. The players appeared to experience posttraumatic growth sometime after their release, consistent with previous research (Neely et al., 2018). The healing process following release has allowed Arthur to develop a new outlook on life and view of his personal world (Athens, 1995). Keith and Arthur poignantly captured how the process of release affected the players' sense of belonging in the world, and candidly demonstrates the existential meaning players placed on their release (Nizza et al., 2021). This extends previous research showing former academy cricket and rugby players developed positive psychological characteristics during their time on the development pathway that supported their transition into other domains following deselection (Knights et al., 2016; Williams and MacNamara, 2020).

## Applied implications

Release from professional academy football can be a traumatic experience and lead to long-term psychological difficulties. Our findings highlight opportunities for players, clubs and key stakeholders to consider how the release process is handled. Throughout the release process, players experienced feelings of a lack of control and often displayed maladaptive coping strategies. Academies could develop pre-release programs aimed at preparing players for release by developing a series of psychosocial skills, which could support their transition out of football. This could include developing coping skills, improving their ability to seek social support, developing their confidence and self-esteem, and increasing autonomy (Williams and MacNamara, 2020).

As part of this preparatory program, previously released players could volunteer to talk to players at professional academies about their experiences of being released, as Arthur suggested in our study. This finding is noteworthy, as the insight and lived experiences of the process from released players would be valuable for current players in preparing them for release, especially with regards to championing their positive experiences of seeking support from counseling services following their release. If there is more realism from players and coaches, accepting the likelihood that becoming a professional is very slim, they may begin to prepare for a potential transition. Interestingly, Keith reported being repeatedly made aware of the high percentage of players who do not become professionals. This realism from the club, although possibly harsh for players to hear, may allow players to begin the appropriate career planning associated with healthy career transition. If the subject of release is avoided by clubs, as with Ben's experience, combined with the fact players suggested they focused all their efforts on becoming a professional footballer, and any thought otherwise was dismissed, the likelihood of a problematic transition increases (Brown and Potrac, 2009).

Through an IPA approach we revealed that players may experience the release process differently. Therefore, an individualized approach to supporting player transition out of academies is warranted. This includes considering the player's perceptions of the potential reasons for their release, and thus their levels of acceptance, with our study suggesting that players who are released through injury or with a long-term injury should be identified as potentially needing greater support. This is supported by previous research, with 11 counselors who work for the PFA suggesting that deselection and forced retirement through injury was traumatic for players (Gervis et al., 2019).

As some players struggled with mental health problems following their release, improvements in provision for player aftercare following release are warranted, including formalized professional support. Recently, Crystal Palace Football Club have announced an aftercare programme to support players released from their academy between the ages of 18–23, which is a vital step forward (Crystal Palace Football Club, 2022). In the present study, Arthur benefitted from PFA counseling sessions once he sought support. However, it is unclear how many released youth footballers are unaware of this service—or are not accessing it. Thus, the benefit of using PFA counseling services could be promoted and encouraged for all released scholars. All released players that are members of the PFA could be scheduled in for an initial counseling session with the PFA's counseling network by their clubs, which the players could continue with if they feel it is beneficial. This supports a recent review calling for clubs to offer released players counseling during the transition away from the club and beyond (Wilkinson, 2021).

## Strengths and limitations

Our study gained valuable insight into the processes involved with release from academy football, being the first study to qualitatively explore players' experiences of the release process. We presented novel findings that: release is a process and individualized to each player, release with a long-term injury can result in more psychological difficulties and in the longer-term players develop positive psychosocial outcomes. This expands current knowledge and understanding in a hard-to-reach population. A further strength was the caliber of participant recruited, and the levels of football they have played at. Nevertheless, a potential limitation was the use of retrospective interviews, due to potential memory decay and a bias around players' memories of their experiences. Yet, these fading effects are reduced regarding momentous events, such as a player's release (Pillemer, 2001). It also allowed the players' growth since their release to be explored, with them reflecting on their release both at the time and retrospectively.

## Future research

One possible direction for future research is exploring the experiences of academy staff members involved in the release process and understanding their perspectives on the process. This may shed further light on how coaches feel they prepare players for release, possibly through foreshadowing their fate and marginalizing players. This would provide further understanding in the area from a different key stakeholder. Further future research should look to explore players' experiences of release “live” at the time of selection procedures. A prospective longitudinal study over an extended period would be a useful avenue for future research, exploring players' perceptions prior to, at the time of, and post release. Furthermore, players' divestment of athletic identity following release, or as in Ben's case, the reinforcement of this identity should be explored further to understand the longer-term impacts of this. As noted in the present study, positive growth and outcomes were highlighted by the players and further study will aid the knowledge of this.

## Conclusion

To our knowledge, this study was the first to qualitatively explore elite youth footballers' experiences of release. Players reported a range of psychosocial effects associated with their release, and limited aftercare following their release, which affected the quality of their transition away from full time football. Through a qualitative, IPA approach, the present study adds in depth knowledge and understanding of both the release process, and the lived experiences of elite youth released footballers, to the literature.

## Data availability statement

The datasets presented in this article are not readily available because it's not possible to share the interview data as the interviews contain information that could be identifiable. Requests to access the datasets should be directed to tom.mcglinchey@ntu.ac.uk.

## Ethics statement

The studies involving human participants were reviewed and approved by the Non-Invasive Ethical Review Committee, School of Science and Technology, Nottingham Trent University. The patients/participants provided their written informed consent to participate in this study.

## Author contributions

TM and CS contributed to the conception and design of the study. TM performed the data collection, performed the initial data analysis, and wrote the first draft of the paper. All authors contributed to manuscript revision, read, and approved the submitted version.

## Funding

Sport, Health, and Performance Enhancement (SHAPE) Research Center, Department of Sport Science, School of Science and Technology, Nottingham Trent University, UK paid the publication fees.

## Conflict of interest

The authors declare that the research was conducted in the absence of any commercial or financial relationships that could be construed as a potential conflict of interest.

## Publisher's note

All claims expressed in this article are solely those of the authors and do not necessarily represent those of their affiliated organizations, or those of the publisher, the editors and the reviewers. Any product that may be evaluated in this article, or claim that may be made by its manufacturer, is not guaranteed or endorsed by the publisher.
